# Phase Angle Is Related to 10 m and 30 m Sprint Time and Repeated-Sprint Ability in Young Male Soccer Players

**DOI:** 10.3390/ijerph18094405

**Published:** 2021-04-21

**Authors:** Priscila Custódio Martins, Anderson Santiago Teixeira, Luiz Guilherme ANTONACCI Guglielmo, Juliana Sabino Francisco, Diego Augusto Santos Silva, Fábio Yuzo Nakamura, Luiz Rodrigo Augustemak de Lima

**Affiliations:** 1 Research Center in Kinanthropometry and Human Performance, Sports Center, Federal University of Santa Catarina, Florianopolis 88040-900, SC, Brazil; julianasabinofrancisco@outlook.com (J.S.F.); diegoaugustoss@yahoo.com.br (D.A.S.S.); 2 Physical Effort Laboratory, Sports Center, Federal University of Santa Catarina, Florianópolis 88040-900, SC, Brazil; anderson.santeixeira@gmail.com (A.S.T.); luiz.guilherme@ufsc.br (L.G.A.G.); 3 Research Group for Development of Football and Futsal, Sports Center, Federal University of Santa Catarina, Florianópolis 88040-900, SC, Brazil; 4 Associate Graduate Program in Physical Education UPE/UFPB, João Pessoa 58051-900, PB, Brazil; fabioy_nakamura@yahoo.com.br; 5 Institute of Physical Education and Sport, Federal University of Alagoas, Maceió 57072-900, AL, Brazil; augustemak@gmail.com

**Keywords:** anaerobic running, bioelectrical impedance, body composition, team sports

## Abstract

Objective: To examine the association between phase angle (PhA) and bioelectrical impedance vector analysis (BIVA) and components of physical performance in male youth soccer players. Design: Cross-sectional. Methods: Sixty-two players from two professional soccer academies were recruited. Electrical bioimpedance was used to obtain the PhA and BIVA. Body fat (BF) and lean soft tissue mass (LSTM) were measured by dual-energy X-ray absorptiometry. All players completed physical tests including the standing long jump (SLJ), Carminatti’s test (peak speed at the end of the test, PS_T-CAR_), 10 m and 30 m straight-line sprints, and repeated-sprint ability (RSA) test (RSAbest and RSAmean times). Results: Adjusting for chronological age, BF, and LSTM, multiple regression analysis outputs showed that PhA remained inversely related to RSAmean (β = −0.362; *p* < 0.001), RSAbest (β = −0.239; *p* = 0.020), 10 m (β = −0.379; *p* = 0.012), and 30 m (β = −0.438; *p* < 0.001) sprint times, while the association with PS_T-CAR_ and SLJ performance were statistically non-significant. In addition, BIVA showed that differences in confidence ellipses were found between athletes in the reference population and the study sample (*p* < 0.05). The tolerance ellipses indicated that the athletes in the present study had more total body water (TCW) and lower proportions of intracellular water (ICW) to extracellular water (ECW). The reference population had more TCW and ICW/ECW. Conclusions: Our results suggest that young soccer players with higher PhA values, indicating better cell integrity and functionality, have better performance in typical anaerobic running activities, such as sprinting speed and RSA performance, adjusted to age and body composition characteristics.

## 1. Introduction

Soccer is a popular sport around the world, often chosen by children and adolescents to start their sport careers [1]. Physiological and physical demands of a soccer match are dissimilar between youth (under 18 years) and senior professional players [1,2]. Young soccer players, on average, cover a total distance during competitions of 5.0 km (Under-13), 6.7 km (Under-15), and 9.0 km (Under-17) [2], while the total distance for professional adults is within 10–12 km [1,2]. Regarding near-maximal or maximal efforts, young players can perform more than 20–30 sprints [2] with a mean duration of 1.4 ± 0.4 s [3]. Soccer also requires several episodes of acceleration, deceleration, and directional changes during a game [3]. These features highlight the importance of soccer-specific physical attributes, such as intermittent endurance running (IER) capacity, muscle power, speed, and repeated-sprint ability (RSA), to support the physical demands required during an official game in adolescent athletes.

Identifying the determinants of physical performance in soccer players is essential to help coaches and conditioning specialists during the planning of training programs. Among the major factors affecting soccer performance are physiological, metabolic, neuromuscular, and anthropometric features [4,5,6,7,8]. For instance, RSA performance has been linked to aerobic fitness parameters [4], neuromuscular abilities [4], and functional movement patterns [6] in soccer players. Buchheit et al. [5] showed that acceleration and maximum sprinting speed are influenced by mechanical properties. Improvements in IER capacity have been positively related to the annual training volume and fat-free mass, but inversely associated with body fat (BF) [9,10]. Although the factors influencing physical performance are well documented, the association between cellular parameters and physical performance measures in team sport settings has not received much attention, especially in youth male soccer players.

Current evidence suggests that phase angle (PhA) might be used as a biological indicator related to cellular integrity and functionality [11,12]. Earlier investigations have shown that PhA is positively associated with muscle mass, strength, and functional capacity in humans [12]. Moreover, training-induced increases in PhA are moderately associated with gains in muscle quality index (defined as 1RM strength per kilogram of lean soft tissue mass (LSTM)) [12]. Among the few studies available in the scientific literature examining the topic PhA and soccer players, Mascherini et al. [13] found significant increases in PhA from the pre-to mid-season period in professional players. The increases in PhA might indicate increases in body cell mass (BCM) and muscle function as a consequence of regular training [13]. Koury et al. [14] also observed that skeletal age and erythrocyte zinc concentration were positively related to PhA in 13–14-year-old male soccer players, accounting for 34% of inter-individual variation. These results reinforce that the possible influence of PhA on physical performance of youth athletes can be partially attributed to the effects of maturation and zinc status [14]. PhA has also been related to chronological age [15] and performance level (elite vs. sub-elite) [16] in soccer players. To date, the study of Nabuco et al. (2019) [17] was the first to identify PhA as a significant predictor of maximum power and fatigue index estimated from an RSA test in 99 adult male soccer players. However, fatigue index is a poorly reliable measure to evaluate soccer players [18]. Thus, in order to better inform researchers and practitioners about the feasibility of the PhA and its degree of relationship with the players’ individual fitness levels, examining whether PhA is related with other more reliable field-based performance indices (e.g., RSA mean time, 10 m and 30 m sprint test) when assessing youth soccer players is needed.

The biological rationale in assuming the PhA as a predictor of motor performance in soccer is based on some primary findings in the literature. First, PhA has been positively associated with BCM [19]. BCM comprises the metabolically active and protein-rich compartments in the body and is one of the predictors of muscle strength production and, in turn, of athletic performance [20]. Indeed, PhA has been associated with 1RM squat and bench press in men trained with resistance, even adjusting for lean mass and body fat percentage [21]. Second, increased PhA values can indicate a better body hydration state, since the higher the PhA, the lower the body resistance (R), thus reflecting higher intracellular water content (ICW) [11]. A study carried out with 202 men and women (20.4 ± 5.2 years) identified that the athletes with the highest PhA values were those with the highest total body water (TBW) values, mainly in the ICW compartment and, consequently, lower extracellular water (ECW) [22]. Prior studies have also shown the importance of PhA and total body water (TBW) status in power- and strength-related performances in athletes [18,19].

Another parameter of the bioelectrical impedance analysis (BIA) is the bioelectrical impedance vector analysis (BIVA) that provides estimates of the hydration status and body composition of the athletes. Studies have observed that after sports competition, there are changes in the tolerance ellipses, with shortening of the vector [15], or even a decline of the vector to the left [16]. These changes may indicate hyper hydration and adequate hydration, respectively.

The present study aimed to examine the association between PhA and BIVA and components of physical performance in male youth soccer players. The novelty of this investigation was the analysis of a cellular integrity and functionality parameter to examine the relationship of PhA with soccer performance-related physical fitness attributes. From a practical perspective, bioimpedance-derived PhA is a simple, quick, non-invasive, and reliable index that might be used to monitor the players’ overall fitness and wellness condition during training phases [13,14], since performing maximal physical performance assessments in athletes on a regular basis is often impractical.

## 2. Methods

### 2.1. Participants

Sixty-two male adolescent players (age: 15.0 ± 1.4 y; weight: 62.7 ± 11.2 kg; height: 172.8 ± 11.6 cm) from two professional soccer academies of the Brazilian National League were recruited to take part in this cross-sectional study. Of the 62 athletes, 14 were Under-13 (U-13), 25 were Under-15 (U-15), and 23 were Under-17 (U-17). At the time of the study, all the participants took part in three to six 60–90 min training sessions per week, in addition to a competitive match, usually on Saturdays. U-13, U-15, and U-17 players spent, on average, 158.8 ± 8.35 min, 391.2 ± 74.93 min, and 460.6 ± 57.19 min of training per week, respectively. Coaches, players, parents, or tutors were informed of the research procedures, requirements, benefits, and risks before giving written informed consent (parents) and assent (players). Participation was voluntary and players could withdraw at any time without any penalty. The research ethics committee of the local university approved this study. The sample size was calculated a posteriori considering type I error (α = 0.05) and type II error (β = 0.80) to identify PhA association with physical performance variables in athletes. The analysis indicated that the sample of 62 athletes allowed finding of associations with an effect size of 0.50 [23]. All calculations were performed using G*Power software version 3.1.9.2 (Universitat Dusselfodorf, Dusselfodorf, Germany).

### 2.2. Procedures

A cross-sectional study design was used to evaluate the association between PhA and physical performance attributes, composed of standing long jump (SLJ), IER capacity, sprinting speed, and RSA in male youth soccer players. The testing procedures were carried out during the last part of the preparation period of the 2018 season. All assessments were completed within a 2-week period. The first week of testing included only body composition assessments, by means of dual-energy X-ray absorptiometry (DXA) and bioelectrical impedance analysis (BIA), in the University’s laboratory. During the second week, two days of the training microcycle were dedicated to the application of the following physical tests with all players: (i) on the first day: SLJ and Carminatti’s test (T-CAR) and (ii) on the second day: sprint test straight and RSA protocol. No other physical training activities were performed on the test days. In addition, 24 h of rest were allowed between each test day to ensure the athletes’ optimal recovery. All physical assessments were carried out on a grass pitch at the club’s own facilities.

### 2.3. Body Composition (Covariates)

Body composition analysis was performed by dual-energy X-ray absorptiometry, Lunar Prodigy Advance, Discovery Wi Fan -Beam -S/N 81,593, (GE, Medical Systems, Madison, WI, USA). Attenuation of X-rays in body tissues was computed by Encore software 13.60.033 pediatric version (GE Lunar Corporation, Madison, WI, USA). The equipment was calibrated daily according to the manufacturer, and phantom calibration was performed weekly. A previously trained investigator performed all the evaluations following standard procedures [24]. During evaluations, participants wore appropriate clothing, and were barefoot and without either earrings or rings. Based on the results obtained, BF (kg) and LSTM (kg) were considered. Since BF and LSTM have been related to PhA [25], both measures were inserted as covariates in the multiple regression analysis.

### 2.4. Phase Angle (Independent Variable)

PhA analysis was performed using electrical bioimpedance (BIA), model InBody 720, octopolar multi-frequency equipment (Biospace, Los Angeles, CA, USA). The BIA model used showed an acceptable reproducibility and accuracy for estimating body composition tissues at a frequency of 50 Hz [26]. In addition, the manufacturer emphasizes in the equipment manual that a high level of accuracy is found when following the correct measurement procedures. The BIA provided data on impedance (Z) and reactance (Xc) from the segmental values (trunk, lower limbs (left and right) and upper limbs (left and right)) of these variables at the frequency of 50 kHz. The resistance values (R) were calculated by the proportional sum of the body, in which the upper limbs represent 40%, the trunk represents 10%, and the lower members represent 50% of the total body R. To calculate the PhA, we used the tangent arc formula (Xc/R) × 180°/π [27]. During the evaluation, participants remained in an orthostatic position, holding two levers, with their feet positioned under a platform. The evaluation lasted approximately two minutes and was performed only once per player. All players were instructed to follow the pre-test recommendations that included: fasting for at least four hours, wearing light clothing, being barefoot, without either earrings or rings or other metals, abstaining from vigorous physical activity on the previous day, and abstaining from drinks with a high-dose of caffeine in the previous 12 h [25,27].

### 2.5. Physical Performance Indices (Dependent Variables)

The SLJ is a commonly used test to measure the explosive strength and power of the lower limbs [28]. The SLJ test was performed starting from a standing position. The jump is evaluated by the horizontal distance from the takeoff line to the mark made by the heel or the nearest point of contact to the takeoff line at landing. The distance in the SLJ was measured to the nearest 0.5 cm using a tape measure. The best attempt of the 3 jumps carried out was used for further analyses. Reliability has provided acceptable levels in young athletes [29].

The IER capacity was evaluated by means of Carminatti’s test (T-CAR) [30]. The test consisted of intermittent shuttle runs of 12 s performed between 2 lines set at progressive distances, with a 6-s recovery between each run and a total stage time of 90 s. The protocol had a starting velocity of 9 km·h^−1^ over a running distance of 30 m (15 m back and forth). The length in a single direction was increased progressively by 1 m at every level. Each stage consisted of 5 repetitions with a 6 s walking period between 2 lines set 2.5 m from the starting line. Eight to 10 athletes were evaluated simultaneously, with the running pace dictated by a pre-recorded audio system. The test ended when participants failed to follow the audio cues on the front line for 2 successive repetitions (objective criteria observed by researchers). The speed in the final stage (PS_T-CAR_) during the T-CAR was retained as the performance criterion. The reliability of PS_T-CAR_ has been established previously [30].

Before the sprint test, players performed a standardized 10 min warm-up of progressive runs and accelerations, administered by the physical coach of each age category. The sprint time was measured to the nearest 0.01 s using two pairs of single-beamed photocells (Speed Test 6.0 CEFISE, Nova Odessa, SP, Brazil). The starting position was standardized to a still split standing position with the toe of the preferred foot forward, 0.5 m behind the starting line. Initially, the first and second pair of photocells were positioned at 0 m (start line) and 10 m distances, respectively. All players sprinted twice for the 10 m distance. After these two initial attempts, the first pair of photocells was kept at 0 m while the second one was now positioned at 30 m distance. Two further two attempts were performed by players. The photocells were set ∼0.7 m above the floor (i.e., hip level) to capture the trunk movement rather than a false trigger from a limb [30,31]. The fastest time in the 10 m and 30 m sprints was retained for the analyses. The reliability of 10 m and 30 m sprint times has been described elsewhere [31,32].

The RSA test consisted of 6 × 40 m (20 + 20 m with a 180° change of direction) sprints separated by 20 s of passive recovery [18]. The players started 0.5 m behind the start line, which was marked by a pair of single-beamed photocells (Speed Test 6.0 CEFISE, Nova Odessa, SP, Brazil). Before starting, the players were instructed to run as fast as possible to the end of the 20 m course, which was marked with 2 cones, then perform a quick change of direction (180°), and run in the direction of the start line. Following each sprint, players decelerated and walked to the starting line in readiness for the subsequent sprint. Five seconds prior to the next sprint, the players assumed the starting position and a 3 s regressive countdown was provided to commence their sprint. The best (RSAbest) and mean sprint times (RSAmean) were recorded as the performance criteria. The reliability of RSAbest and RSAmean has been described elsewhere [18,29].

### 2.6. Statistical Analysis

Descriptive statistics (mean ± SD) were calculated for the total sample and for competitive age groups (U-13, U-15, and U-17). Normality was checked using the Kolmogorov–Smirnov test. Between-age group differences were tested using univariate analysis of variance (ANOVA). The magnitude of the differences was assessed using standardized mean differences (Cohen’s *d* effect size, ES) with thresholds of 0.20, 0.60, 1.20, 2.0, and 4.0 for small, moderate, large, very large, and extremely large [33]. Pearson’s product-moment correlations were calculated to verify the relationship between PhA (independent variable) and physical performance outcomes (dependent variables). Partial correlations controlling for age, LSTM, and BF were also calculated to examine the association between R and Xc with physical performance outcomes. These partial correlation analyses were performed to identify which of the variables (R or Xc) of PhA are more related to the physical performance measures. The following criteria were adopted for interpreting the magnitude of correlation (r) between test measures: ≤0.1 trivial, >0.1–0.3 small, >0.3–0.5 moderate, >0.5–0.7 large, >0.7–0.9 very large, and >0.9–1.0 almost perfect [33]. Finally, multiple linear regression analysis was used to test the association between PhA and physical performance measures, adjusting for control variables. Three different adjusted-models were tested: (1) age; (2) age and LSTM; and (3) age, LSTM, and BF. Regression coefficients (β), 95% confidence interval, and determination coefficient were estimated for each model analyzed (adjusted R^2^). For all analyses, STATA software (StataCorp LLC, College Station, TX, USA), version 14.0 was used, establishing *p* ≤ 0.05.

For confidence ellipses, the values of R and Xc were standardized by height (meters) and the differences between the reference population of Toselli et al. (2020) [34] and the sample of the present study were analyzed using the Hotelling T^2^ test [35]. The Hotelling T^2^ test was created to compare vectors of population means [34]. For the tolerance ellipse, the values of R and Xc standardized by height were expressed. All analyses were performed using BIVA 2002 software (Microsoft, Padova, Italy), establishing a value of *p* ≤ 0.05.

## 3. Results

Descriptive data for the total sample and age categories (U-13, U-15, and U-17) are summarized in Table 1. Age-related differences were found for all variables investigated (*p* < 0.05), with the exception of BF(body fat). U-17 players were older, heavier, taller, and had higher reactance, PhA, PS_T-CAR_, 30 m sprint time, RSAmean, and RSAbest than their U-13 and U-15 teammates (*p* < 0.01).

**Figure 1:**
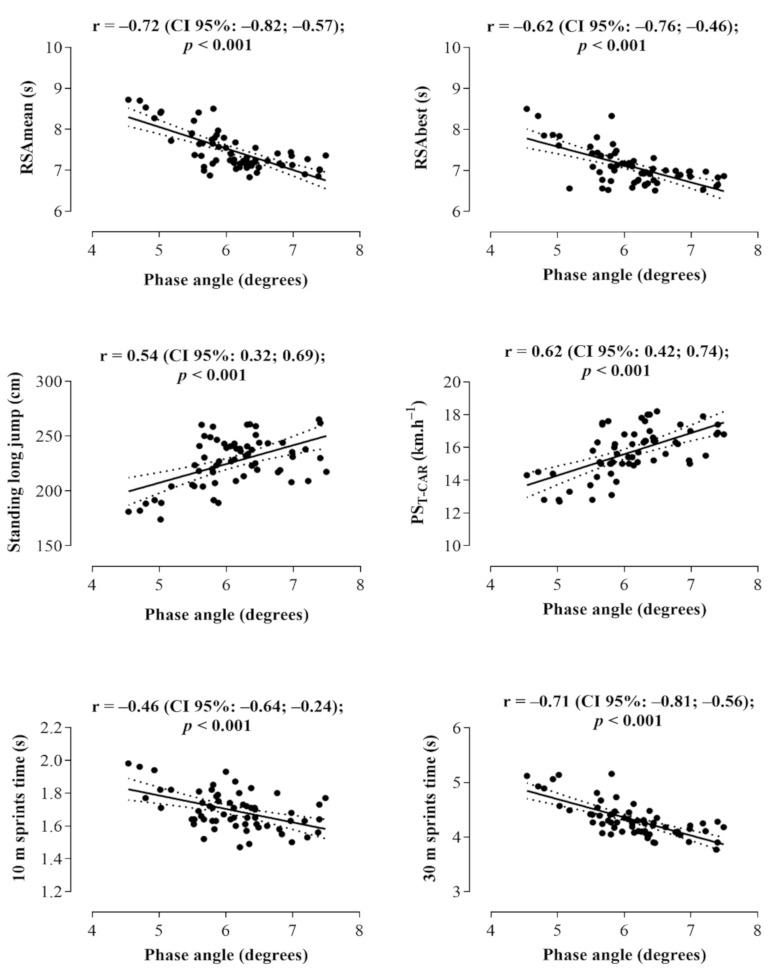
Correlation between the phase angle and physical performance outcomes for total sample (*n* = 62).

For the total sample (*n* = 62), correlation analyses showed that PhA was positively related to the SLJ and PS_T-CAR_ performances in young soccer athletes, while PhA was inversely related to the RSAmean, RSAbest, 10 m, and 30 m sprint times (Figure 1). Partial correlation analyses identified that Xc was not related to any physical performance attributes (*p* > 0.05), while R was significantly associated only with RSAmean (r = 0.24; *p* = 0.05), 10 m (r = 0.35; *p* = 0.01), and 30 m (r = 0.29; *p* = 0.03) sprint times.

**Figure 2:**
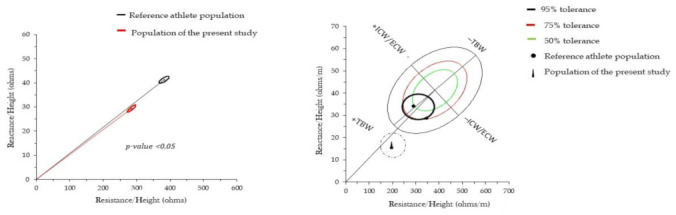
Although phase angle (PhA) was directly associated with the standing long jump (SLJ) (R^2^ = 0.272, *p* < 0.001) in the crude model, this association was statistically non-significant after adjusting for age, LSTM, and body fat (BF) (R^2^ = 0.531, *p* = 0.109). In addition, the PhA was directly associated with PS_T-CAR_ (R^2^ = 0.360, *p* < 0.001) in the crude and age-adjusted models, accounting for 70% of the variance in the PS_T-CAR_. PhA was not a significant explanatory variable of the PS_T-CAR_ after adjusting for LSTM and BF (R^2^ = 0.703, *p* = 0.169). PhA was inversely associated with the 10 m and 30 m sprint times in the crude and age, LSTM, and BF-adjusted models, accounting for 24% and 61% of the variability in 10 m and 30 m sprints time, respectively. PhA was also inversely associated with RSAmean and RSAbest times, in which the models composed by age, LSTM, and BF explained approximately 74% and 64% of the variability in these anaerobic physical performance components, respectively (Table 2).

The BIVA analyses showed that there were differences in the confidence ellipses between the athletes of the reference population [34] and the study sample (*p* < 0.05). The tolerance ellipses demonstrated that the athletes in the present study had more TBW and less AIC/AEC ratio (Figure 2). The reference population had more TBW and ICW/ECW. The bioimpedance values measured for the entire sample of athletes in the present study were R/height = 284.72.1 (standard deviation ± 40.53) and Xc/height = 41.3 (standard deviation ± 4.70).

## 4. Discussion

The current study examined the association between PhA and BIVA and soccer-related physical performance attributes in male youth soccer players. The novelty of this study were the significant associations of PhA with IER capacity, SLJ, 10 m and 30 m sprint times, and RSA performance in youth soccer players, highlighting the biological role of PhA in influencing physical performance. PhA also remained a significant predictor of 10 m and 30 m sprint times and RSA performance outcomes after adjustment for age and body composition components, such as LSTM and BF. To date, this is the first study to associate PhA with soccer-related physical performance attributes in adolescent players involved in an organized and systematic training program, providing unique insights into the topic.

PhA is a widely explored cellular health marker in clinical and interventional studies [12] and pediatric populations [36,37]. On the other hand, the potential role of PhA in physical performance outcomes in youth athletes is still poorly understood. In our sample of soccer players, age-related differences were found for PhA, with U-13 players (5.2 ± 0.4°) displaying significantly lower PhA values than U-15 (6.2 ± 0.4°) and U-17 (6.5 ± 0.5°) players. This finding is in agreement with previous investigations showing positive associations between PhA and age during childhood and adolescence periods [36,37]. With growth and maturation, the relative contribution of protein and minerals to the LSTM compartment increases, which could explain the gains in PhA during adolescence [28], since PhA has been directly related to BCM [19]. Furthermore, LSTM is recognized as a body composition compartment that contains a large amount of water and electrolytes [25]. Thus, the age-related increases in ICW and decreases in R, due to these increases in protein content and, in turn, in LSTM, also result in better conductivity, contributing to improved PhA during adolescence [14]. The mean PhA values found in this study were slightly lower than those reported in prior studies with age-matched male athletes (U-13–U-17: range = 6.3–7.0°) [14,37] and non-athlete adolescents (U-11–U-14: range = 5.8–6.1°; U-15–U-17: range = 6.8–7.5°) [36].

In the present study, regression analysis outputs identified PhA as a significant predictor of intermittent endurance performance (PS_T-CAR_), accounting for 36% of inter-individual variance. The inclusion of age increased the explained variance to 70% (model 2). These findings highlight that a better cellular integrity and functionality profile, indicated by a higher PhA value, could be relevant to performance and repeated intermittent high-intensity efforts regardless of age-related improvements that occur over the pubertal years. However, the influence of PhA on intermittent endurance performance disappeared when LSTM (model 3) and BF (model 4) were accounted for. This latter result suggests that the positive relationship between PhA and PS_T-CAR_ in adolescent soccer players seems to be mediated by the amount of skeletal muscle and BF.

Current available evidence has also indicated PhA as an important measure related to muscle strength [21], with applicability to monitoring training-induced adaptations in the neuromuscular system in the elderly population [12]. It is unknown whether this cellular integrity and functionality parameter is also related to muscle performance during the pubertal years. In the present study, a positive association was found between PhA and SLJ performance, possibly due to its relationship with neuromuscular function [21]. Nonetheless, when age, LSTM and BF, and models 1, 2, and 3, respectively were inserted as covariates, there was no association of the PhA with neuromuscular performance in our sample of adolescent soccer players. These data potentially indicate that age and body composition indicators are stronger predictors of SLJ performance than the PhA measure during the puberty period. Furthermore, SLJ performance can also be influenced by the athlete’s inter-limb motor coordination to execute the jump movement as far forward as possible [7,8]. In a longitudinal mixed study, Deprez et al. [8] demonstrated that age, body size (given by leg length), body composition (fat-free mass derived from a two-component model), flexibility (sit-and-reach), and motor coordination (Körperkoordinationstest für Kinder, KTK) were among the main predictors of explosive muscle power in 356 Flemish youth soccer players. These factors could provide a theoretical framework to explain the lack of association between PhA and neuromuscular performance evaluated by means of SLJ in young soccer players.

The most interesting finding in our study was that greater cell integrity and functionality, indicated by a higher PhA, was significantly associated with soccer-related anaerobic physical attributes, such as 10 m and 30 m sprint time and RSA outputs, even after controlling for age and body composition indicators. Particularly for sprint performance, we found that PhA was only moderately related to 10 m sprint time, but very largely correlated with 30 m sprint time. To the best of our knowledge, there are no clear assumptions in the literature in terms of mechanisms to justify this stronger relationship noticed between PhA and 30 m sprint time. A possible partial explanation could be the greater inter-individual variability (SD: 0.3 vs. 0.1 s; coefficient of variation: 7.33% vs. 6.74%) found for the 30 m than 10 m sprint time, respectively. As short and long distances sprint actions are solicited in various decisive moments of the game (e.g., goal scoring) [38], our findings show that PhA emerges as a promising complementary measure to help monitor the player’s readiness status to compete or to train. In this study, players with higher PhA values displayed improved running anaerobic performance. In a recent study, Nabuco et al. [17] showed a moderate association between PhA and estimated maximum power (positive) and the fatigue index (negative) derived from a running anaerobic sprint test (RAST) in professional soccer players. An additional innovative contribution of our study to that of Nabuco et al. [17] was the use of different soccer-related anaerobic running measures which are more reproducible than the fatigue index [18]. Our finding is also similar to that reported by Pollastri et al. [39], showing a positive association between PhA and mean power output of short duration efforts (i.e., 15 s) in elite adult cycling athletes. Although the samples and sport modalities were divergent (adolescent vs. adult and soccer vs. cycling), these results provide primary evidence, highlighting the role of the PhA as a determinant of physical performance in adolescent and adult athletes.

It has been shown that PhA is an objective indicator of cellular health, with higher values reflecting better cellularity, cell membrane integrity, and cell function, while lower PhA values can indicate decreased cell integrity [40]. Considering that PhA is a measure derived from R and Xc [11], any alteration in cellular membrane integrity (Xc), body fluid (R), or a combination of both, results in changes in PhA. In this study, partial correlation analyses controlling for confounder variables (i.e., age and body composition indicators) identified that R was significantly associated with RSAmean (r = 0.24; *p* = 0.050), 10 m (r = 0.35; *p* = 0.006), and 30 m (r = 0.29; *p* = 0.026) sprint times, while Xc was not related to any maximal running sprint performance outcomes. Considering that in the human body R reflects the hydration of body tissue and is inversely proportional to the intracellular fluids [11], the association found between PhA and sprinting and RSA performances in our study may potentially be linked to the level of R of body tissues and, in turn, to the athlete’s cellular hydration state. The small-to-very large correlations reported above support the proposal that R affects performance in running activities that require maximum speed and power production. Thus, it can be partially suggested that a higher human body R and, in turn, a lower PhA, results in decreased anaerobic running performance, possibly due to the lower intracellular water content. Supporting our findings, a prior study showed that negative changes in body fluid content (e.g., reductions in total body and intracellular water) can impair muscle power output in adult judo athletes [41].

In the present study, BIVA has shown that there were differences in confidence ellipses between athletes in the reference population [34] and the study sample (*p*-value < 0.05). The tolerance ellipse showed that the athletes in the present study had more total body water (TBW) and less proportion of ICW to extracellular water ECW. The reference population had more TBW and ICW/ECW. Tosseli et al. (2020) [34] evaluated youth soccer players with different maturation levels (70.8% of the players were classified as “punctual”, 16.3% as “earlier”, and 12.9% as “later”) for somatic maturation, within each age category. The authors observed that TBW was higher (*p* < 0.01) in athletes classified as “early” maturity status compared to the other two groups and the classic BIVA confirmed these results. Campa et al. (2020) [21] evaluated soccer athletes and identified that vector displacements in athletes from the upper right to the lower left, aiming at the 50th percentile on the R-Xc graph, where the U-19 group was positioned exactly in the center of the ellipse, showed a BCM similar to that of the 219 soccer players in the first Italian division measured by Micheli et al. [26]. In addition, the authors identified that the players’ average impedance vectors divided by chronological age indicated that from the Under-15 category, the athletes were positioned within the tolerance ellipses.

A strong point of our study is the analysis of the impact of PhA on crucial soccer-related physical performance attributes in well-trained adolescent soccer players, along with the inclusion of precise and valid measures of body composition measurements as covariates that may influence these associations.

This study is not without its limitations. First, no biological maturation indicator was considered in this study. A recent study reported that skeletal maturity status should be taken into account in the interpretation of the PhA in youth soccer players [15]. However, it should be recognized that determining skeletal age is costly and requires specialized equipment and interpretation, which may hinder its use in some field conditions. Second, some mineral nutrients such as zinc and magnesium, which play a key role in this cellular health parameter (PhA), were not evaluated [42]. Third, the data found in our study cannot be extrapolated to other populations. Finally, in the current study, training load metrics were not quantified during the microcycle in which the evaluations were carried out. The great variability of BIA instruments can be considered a limitation of the method; however, the use of crude measures, such as resistance, reactance, PhA, and BIVA data can minimize errors, although it is necessary to observe the characteristics of each model of the instrument to avoid misinterpretation [43].

## 5. Conclusions

This study concludes that PhA is associated with 10 m and 30 m sprint times and RSA performance in young male soccer players regardless of age-related variability and body composition measures. These data could contribute to better understanding of the interaction between PhA, a non-invasive indicator of cellular health, and physical performance attributes in adolescent soccer players. Future studies are suggested to investigate seasonal changes in PhA and body fluids over an entire competitive season and to establish possible relations with the training load placed on soccer players. In addition, this study presented BIVA data and observed that the athletes in the present study showed differences compared to the athletes in the reference population.

## 6. What Does This Article Add?

- Physical assessments requiring the application of maximum efforts from players are not always possible to be implemented over the season in order to monitor the players’ physical readiness state. From our findings, PhA emerge as a non-invasive cellular health marker obtained at rest condition to be considered in the context of screening tools used in adolescent athletes due to its relationship with crucial soccer-specific anaerobic running activities such as sprinting speed and RSA.
- Our results contribute to the body of knowledge produced to date on this topic, addressing the practical application of this measurement (i.e., PhA) within youth sports settings.

## Figures and Tables

**Table 1:** Descriptive statistics (mean ± SD) for body composition and physical performance outcomes considering total sample (*n* = 62) and age categories (U-13, U-15, and U-17) with ANOVA outputs and effect sizes (with 95% confidence interval).

	Total (*n* = 62)	U-13 [1] (*n* = 14)	U-15 [2] (*n* = 25)	U-17 [3] (*n* = 23)	ANOVA		Effect Size (95%CI)		
	Mean (SD)	Mean (SD)	Mean (SD)	Mean (SD)	F	*p*	3 vs. 1	3 vs. 2	2 vs. 1
Age (years)	15.0 (1.4)	12.7 (0.2)	14.9 (0.2)	16.5 (0.5)	-	-	-	-	-
Body mass (kg)	62.7 (11.2)	45.5 (6.3) * †	65.8 (6.4) §	69.7 (5.2)	77.2	<0.01	4.30 (3.04; 5.37) Very large	0.67 (0.07; 1.24) Moderate	3.19 (2.17; 4.08) Very large
Height (cm)	172.8 (11.6)	155.6 (6.6) * †	175.9 (7.2)	179.8 (6.0)	61.8	<0.01	3.80 (2.71; 4.89) Very large	0.59 (0.00; 1.15) Small	2.90 (1.93; 3.75) Very large
Body fat (kg)	7.7 (2.7)	7.5 (1.6)	8.2 (3.0)	7.2 (2.8)	0.8	0.20	−0.12 (−0.79; 0.54) Trivial	−0.34 (−0.91; 0.23) Small	0.27 (−0.39; 0.92) Small
LSTM (kg)	52.6 (10.1)	38.2 (8.5) †	54.9 (4.3)	58.9 (6.5)	49.9	0.01	2.83 (1.86; 3.69) Very large	0.73 (0.14; 1.30) Moderate	2.73 (1.79; 3.56) Very large
Resistance (ohms)	485.6 (47.1)	518.0 (54.3) *	471.6 (40.7)	481.0 (41.1)	5.1	0.03	−0.80 (−1.47; −0.09) Moderate	0.23 (−0.34; 0.79) Small	−1.01 (−1.68; −0.30) Moderate
Reactance (ohms)	51.4 (5.4)	47.4 (3.0) †	50.9 (4.1) §	54.6 (6.0)	10.0	<0.01	1.41 (0.65; 2.12) Large	0.73 (0.13; 1.30) Moderate	0.93 (0.23; 1.60) Moderate
Phase angle (degrees)	6.1 (0.6)	5.2 (0.4) * †	6.2 (0.4)	6.5 (0.6)	26.8	<0.01	2.43 (1.52; 3.24) Very Large	0.59 (0.01; 1.16) Small	2.50 (1.60; 3.30) Very large
Standing long jump (cm)	226.9 (22.6)	194.4 (11.7) * †	233.2 (14.6)	239.4 (15.2)	52.7	<0.01	3.21 (2.17; 4.12) Very Large	0.42 (−0.16; 0.98) Small	2.84 (1.88; 3.68) Very large
10 m sprint time (s)	1.7 (0.1)	1.8 (0.1) * †	1.7 (0.1)	1.6 (0.1)	5.4	<0.01	−2.00 (−2.76; −1.16) Large	−1.00 (−1.58; −0.38) Moderate	−1.00 (−1.67; −0.29) Moderate
30 m sprint time (s)	4.3 (0.3)	4.8 (0.3) * †	4.2 (0.2)	4.1 (0.2)	45.1	<0.01	−2.89 (−3.76; −1.90) Very large	−0.50 (−1.07; 0.08) Small	−2.50 (−3.30; −1.60) Very large
PS_T-CAR_ (km·h^−1^)	15.7 (1.4)	13.7 (0.8) * †	15.6 (0.7) §	17.0 (0.6)	88.3	<0.01	4.84 (3.47; 6.01) Very large	2.14 (1.40; 2.81) Very large	2.58 (1.66; 3.39) Very large
RSAmean (s)	7.4 (0.5)	8.2 (0.3) *†	7.3 (0.2) §	7.1 (0.2)	83.6	<0.01	−4.55 (−5.66; −3.24) Very large	−1.00 (−1.58; −0.38) Moderate	−3.75 (−4.72; −2.63) Very large
RSAbest (s)	7.0 (0.4)	7.7 (0.4) *†	7.0 (0.1) §	6.7 (0.2)	50.9	<0.01	−3.44 (−4.38; −2.35) Very large	−1.92 (−2.57; −1.21) Large	−2.80 (−3.63; −1.85) Very large

LSTM: lean soft tissue mass; PS_T-CAR_: peak velocity derived from Carminatti’s test; RSA: repeated sprint ability; * differences between U−13 and U-15 (*p* < 0.01); † differences between U-13 and U-17 (*p* < 0.01); § differences between U-15 and U-17 (*p* < 0.01). RSAmean: mean time obtained from repeated sprint ability test; RSAbest: best time obtained from repeated sprint ability test.

**Table 2:** Simple and multiple regression analysis outputs between phase angle and physical performance outcomes in young soccer players (*n* = 62).

	β (95%CI)	β Standardized	Adjusted R^2^	*p*
Standing long jump				
Phase angle **	17.717 (10.464; 24.970)	0.533	0.272	<0.001
Model 1	5.485 (4.778; 19.227)	0.165	0.463	0.181
Model 2	5.093 (−2.983; 13.171)	0.153	0.470	0.212
Model 3	6.217 (−1.418; 13.853)	0.187	0.531	0.109
10 m sprint time				
Phase angle **	−0.799 (−0.119; −0.404)	−0.466	0.204	<0.001
Model 1 *	−0.060 (−0.110; −0.009)	−0.351	0.211	0.021
Model 2 *	−0.059 (−0.111; −0.008)	−0.349	0.201	0.023
Model 3 *	−0.065 (−0.115; −0.014)	−0.379	0.240	0.012
30 m sprint time				
Phase angle **	−0.331 (−0.416; −0.247)	−0.712	0.499	<0.001
Model 1 **	−0.206 (−0.305; −0.108)	−0.443	0.598	<0.001
Model 2 **	−0.200 (−0.296; −0.103)	−0.429	0.615	<0.001
Model 3 **	−0.204 (−0.301; −0.107)	−0.438	0.614	<0.001
PS_T-CAR_				
Phase angle **	1.295 (0.859; 1730)	0.609	0.360	<0.001
Model 1 **	0.263 (−0.123; 0.650)	0.124	0.701	<0.001
Model 2	0.248 (−0.139; 0.635)	0.116	0.702	0.205
Model 3	0.270 (0.118; 0.658)	0.116	0.703	0.169
RSAmean				
Phase angle **	−0.523 (−0.654; −0.392)	−0.718	0.508	<0.001
Model 1 **	−0.236 (−0.362; −0.110)	−0.324	0.731	<0.001
Model 2 **	−0.229 (−0.354; −0.104)	−0.314	0.738	0.001
Model 3 **	−0.238 (−0.362; −0.113)	−0.362	0.742	<0.001
RSAbest				
Phase angle **	−0.434 (−0.568; −0.300)	−0.641	0.401	<0.001
Model 1 *	−0.154 (−0.288; −0.205)	−0.228	0.651	0.025
Model 2 *	−0.154 (−0.289; −0.018)	−0.227	0.640	0.027
Model 3 *	−0.162 (−0.298; −0.026)	−0.239	0.644	0.020

Value “*p*” in the table corresponds to the phase angle in the raw and adjusted model. * Significant model (*p* < 0.05); ** significant model (*p* ≤ 0.001); Model 1: phase angle adjusted by the age; Model 2: phase angle adjusted by the age and lean soft tissue mass; Model 3: phase angle adjusted by the age, lean soft tissue mass, and absolute body fat.

## Data Availability

The study did not report any data.

## References

[1] Stølen T., Chamari K., Castagna C., Wisløff U. (2005). Physiology of Soccer: An update. Sports Med..

[2] Vieira L.H.P., Aquino R., Moura F.A., De Barros R.M., Arpini V.M., Oliveira L.P., Bedo B.L., Santiago P.R. (2019). Team dynamics, running, and skill-related performances of Brazilian U11 to professional soccer players during official matches. J. Strength Cond. Res..

[3] Rebelo A., Brito J., Seabra A., Oliveira J., Krustrup P. (2014). Physical match performance of youth football players in relation to physical capacity. Eur. J. Sport Sci..

[4] Baldi M., DASilva J.F., Buzzachera C.F., Castagna C., Guglielmo L.G. (2017). Repeated sprint ability in soccer players: Associations with physiological and neuromuscular factors. J. Sports Med. Phys. Fit..

[5] Buchheit M., Samozino P., Glynn J.A., Michael B.S., Al Haddad H., Mendez-Villanueva A., Morin J.B. (2014). Mechanical determinants of acceleration and maximal sprinting speed in highly trained young soccer players. J. Sports Sci..

[6] Campa F., Semprini G., Júdice P.B., Messina G., Toselli S. (2019). Anthropometry, Physical and Movement Features, and Repeated-sprint Ability in Soccer Players. Int. J. Sports Med..

[7] Deprez D., Valente-Dos-Santos J., Coelho-E-Silva M.J., Lenoir M., Philippaerts R., Vaeyens R. (2015). Longitudinal Development of Explosive Leg Power from Childhood to Adulthood in Soccer Players. Int. J. Sports Med..

[8] Deprez D., Valente-dos-Santos J., Coelho-e-Silva M.J., Lenoir M., Philippaerts R., Vaeyens R. (2015). Multilevel Development Models of Explosive Leg Power in High-Level Soccer Players. Med. Sci. Sports Exerc..

[9] Deprez D., Valente-dos-santos J., Coelho M., Lenoir M., Philippaerts R.M., Vaeyens R. (2014). Modeling Developmental Changes in the Yo-Yo Intermittent Recovery Test Level 1 in Elite Pubertal Soccer Players. Int. J. Sports Physiol. Perform..

[10] Valente-Dos-Santos J., Coelho-e-silva M.J., Duarte J., Figueiredo A.J., Liparotti J.R., Sherar L.B., Elferink-Gemser M.T., Malina R.M. (2012). Longitudinal predictors of aerobic performance in adolescent soccer players. Medicina.

[11] Marini E., Campa F., Buffa R., Stagi S., Matias C.N., Toselli S., Sardinha L.B., Silva A.M. (2020). Phase angle and bioelectrical impedance vector analysis in the evaluation of body composition in athletes. Clin. Nutr..

[12] Sardinha L.B. (2018). Physiology of exercise and phase angle: Another look at BIA. Eur. J. Clin. Nutr..

[13] Mascherini G., Gatterer H., Lukaski H., Burtscher M., Galanti G. (2015). Changes in hydration, body-cell mass and endurance performance of professional soccer players through a competitive season. J. Sports Med. Phys. Fit..

[14] Koury J.C., Trugo N.M.F., Torres A.G. (2014). Phase angle and bioelectrical impedance vectors in adolescent and adult male athletes. Int. J. Sports Physiol. Perform..

[15] Campa F., Silva A.M., Iannuzzi V., Mascherini G., Benedetti L., Toselli S. (2019). The role of somatic maturation on bioimpedance patterns and body composition in male elite youth soccer players. Int. J. Environ. Res. Public Health.

[16] Micheli M.L., Pagani L., Marella M., Gulisano M., Piccoli A., Angelini F., Burtscher M., Gatterer H. (2014). Bioimpedance and im-pedance vector patterns as predictors of league level in male soccer players. Int. J. Sports Physiol. Perform..

[17] Nabuco H.C.G., Silva A.M., Sardinha L.B., Rodrigues F.B., Tomeleri C.M., Ravagnani F.C.P., Cyrino E.S., Ravagnani C.F.C. (2019). Phase Angle is Moderately Associated with Short-term Maximal Intensity Efforts in Soccer Players. Int. J. Sports Med..

[18] Impellizzeri F.M., Rampinini E., Castagna C., Bishop D., Bravo D.F., Tibaudi A., Wisloff U. (2008). Validity of a re-peated-sprint test for football. Int. J. Sports Med..

[19] Melchiorri G., Viero V., Sorge R., Triossi T., Campagna A., Volpe S.L., Lecis D., Tancredi V., Andreoli A. (2017). Body composition analysis to study long-term training effects in elite male water polo athletes. J. Sports Med. Phys. Fit..

[20] Andreoli A., Melchiorri G., Brozzi M., Di Marco A., Volpe S.L., Garofano P., Di Daniele N., De Lorenzo A. (2003). Effect of different sports on body cell mass in highly trained athletes. Acta Diabetol..

[21] Campa F., Matias C.N.M., Teixeira F.J., Reis J.F., Valamatos M.J., Toselli S., Monteiro C.P. (2020). Leucine metabolites do not induce changes in phase angle, bioimpedance vector analysis patterns, and strength in resistance trained men. Appl. Physiol. Nutr. Metab..

[22] Francisco R., Matias C.N., Santos D.A., Campa F., Minderico C.S., Rocha P., Heymsfield S.B., Lukaski H., Sardinha L.B., Silva A.M. (2020). The Predictive Role of Raw Bioelectrical Impedance Parameters in Water Compartments and Fluid Distribution Assessed by Dilution Techniques in Athletes. Int. J. Environ. Res. Public Health.

[23] Crabtree N.J., Arabi A., Bachrach L.K., Fewtrell M., Fuleihan G.E.-H., Kecskemethy H.H., Jaworski M., Gordon C.M. (2014). Dual-Energy X-Ray Absorptiometry Interpretation and Reporting in Children and Adolescents: The Revised 2013 ISCD Pediatric Official Positions. J. Clin. Densitom..

[24] Crabtree N., Kibirige M., Fordham J., Banks L., Muntoni F., Chinn D., Boivin C., Shaw N. (2004). The relationship between lean body mass and bone mineral content in paediatric health and disease. Bone.

[25] Norman K., Stobäus N., Pirlich M., Bosy-Westphal A. (2012). Bioelectrical phase angle and impedance vector analysis—Clinical relevance and applicability of impedance parameters. Clin. Nutr..

[26] De Castro J.A.C., De Lima L.R.A., Silva D.A.S. (2018). Accuracy of octa-polar bioelectrical impedance analysis for the assessment of total and appendicular body composition in children and adolescents with HIV: Comparison with dual ener-gy X-ray absorptiometry and air displacement plethysmography. J. Hum. Nutr. Diet..

[27] Kyle U.G. (2004). Bioelectrical impedance analysis?part I: Review of principles and methods. Clin. Nutr..

[28] Malina R.M., Bouchard C. (1992). Growth, Maturation, and Physical Activity. Med. Sci. Sports Exerc..

[29] Moya-Ramon M., Nakamura F.Y., Teixeira A.S., Granacher U., Santos-Rosa F.J., Sanz-Rivas D., Fernandez-Fernandez J. (2020). Effects of Resisted vs. Conventional Sprint Training on Physical Fitness in Young Elite Tennis Players. J. Hum. Kinet..

[30] Teixeira A.S., da Silva J.F., Carminatti L.J., Dittrich N., Castagna C., Guglielmo L.G. (2014). Reliability and Validity of the Carminatti’s Test for Aerobic Fitness in Youth Soccer Players. J. Strength Cond. Res..

[31] Haugen T., Buchheit M. (2015). Sprint Running Performance Monitoring: Methodological and Practical Considerations. Sports Med..

[32] Altmann S., Spielmann M., Engel F.A., Neumann R., Ringhof S., Oriwol D., Haertel S. (2017). Validity of Single-Beam Timing Lights at Different Heights. J. Strength Cond. Res..

[33] Hopkins W.G., Marshall S.W., Batterham A.M., Hanin J. (2009). Progressive Statistics for Studies in Sports Medicine and Exercise Science. Med. Sci. Sports Exerc..

[34] Toselli S., Marini E., Latessa P.M., Benedetti L., Campa F. (2020). Maturity Related Differences in Body Composition Assessed by Classic and Specific Bioimpedance Vector Analysis among Male Elite Youth Soccer Players. Int. J. Environ. Res. Public Health.

[35] Hotelling H. (1947). Multivariate Quality Control. Techniques of Statistical Analysis.

[36] Mathias-Genovez M.G., Oliveira C.C., Camelo J.S., Del Ciampo L.A., Monteiro J.P. (2015). Bioelectrical Impedance of Vectorial Analysis and Phase Angle in Adolescents. J. Am. Coll. Nutr..

[37] Koury J.C., De Oliveira-Junior A.V., Portugal M.R.C., Oliveira K.D.J.F.D., Donangelo C.M. (2018). Bioimpedance parameters in adolescent athletes in relation to bone maturity and biochemical zinc indices. J. Trace Elements Med. Biol..

[38] Faude O., Koch T., Meyer T. (2012). Straight sprinting is the most frequent action in goal situations in professional foot-ball. J. Sports Sci..

[39] Pollastri L., Lanfranconi F., Tredici G., Burtscher M., Gatterer H. (2015). Body Water Status and Short-term Maximal Power Output during a Multistage Road Bicycle Race (Giro d’Italia 2014). Int. J. Sports Med..

[40] De Lorenzo A., Andreoli A., Matthie J., Withers P. (1997). Predicting body cell mass with bioimpedance by using theoretical methods: A technological review. J. Appl. Physiol..

[41] Silva A., Fields D.A., Heymsfield S.B., Sardinha L.B. (2010). Body Composition and Power Changes in Elite Judo Athletes. Int. J. Sports Med..

[42] Matias C.N., Monteiro C.P., Santos D.A., Martins F., Silva A.M., Laires M.J., Sardinha L.B. (2015). Magnesium and phase angle: A prognostic tool for monitoring cellular integrity in judo athletes. Magnes. Res..

[43] Silva A.M., Matias C.N., Nunes C.L., Santos D.A., Marini E., Lukaski H.C., Sardinha L.B. (2019). Lack of agreement of in vivo raw bioimpedance measurements obtained from two single and multi-frequency bioelectrical impedance devic-es. Eur. J. Clin. Nutr..

